# *O*-GlcNAc-Modification of NSL3 at Thr755 Site Maintains the Holoenzyme Activity of MOF/NSL Histone Acetyltransferase Complex

**DOI:** 10.3390/ijms21010173

**Published:** 2019-12-25

**Authors:** Linhong Zhao, Min Li, Tao Wei, Chang Feng, Tingting Wu, Junaid Ali Shah, Hongsen Liu, Fei Wang, Yong Cai, Jingji Jin

**Affiliations:** 1 School of Life Sciences, Jilin University, Changchun City, Jilin 130012, China; zhaolh16@mails.jlu.edu.cn (L.Z.); minli17@mails.jlu.edu.cn (M.L.); taowei16@mails.jlu.edu.cn (T.W.); fengchang18@mails.jlu.edu.cn (C.F.); ttwu18@mails.jlu.edu.cn (T.W.); junaid1316@mails.jlu.edu.cn (J.A.S.); liuhs17@mails.jlu.edu.cn (H.L.); fei@jlu.edu.cn (F.W.); 2 School of Pharmacy, Changchun University of Chinese Medicine, Changchun City, Jilin 130117, China

**Keywords:** *O*-GlcNAc-modification, nonspecific lethal protein NSL3, histone acetyltransferase, ubiquitin, protein degradation

## Abstract

Both OGT1 (*O*-linked β-N-acetylglucosamine (*O*-GlcNAc) transferase isoform 1) and NSL3 (nonspecific lethal protein 3) are crucial components of the MOF (males absent on the first)/NSL histone acetyltransferase complex. We previously described how global histone H4 acetylation levels were modulated by OGT1/*O*-GlcNAcylation-mediated NSL3 stability. However, the specific modification site of NSL3 and its molecular mechanism of protein stability remain unknown. Here, we present evidence from biochemical experiments arguing that *O*-GlcNAcylation of NSL3 at Thr755 is tightly associated with holoenzyme activity of the MOF/NSL complex. Using in vitro *O*-GlcNAc-transferase assays combined with mass spectrometry, we suppose that the residue Thr755 on NSL3 C-terminus is the major site *O*-GlcNAc-modified by OGT1. Importantly, *O*-GlcNAcylation of this site is involved in the regulation of the ubiquitin-degradation of NSL3, because this site mutation (T755A) promotes the ubiquitin-mediated degradation of NSL3. Further in-depth research found that ubiquitin conjugating enzyme E2 S (UBE2S) accelerated the degradation of NSL3 via direct binding to it. Interestingly, OGT1 and UBE2S competitively bind to NSL3, suggesting the coordination of OGT1–UBE2S in regulating NSL3 stability. Furthermore, *O*-GlcNAcylation of NSL3 Thr755 site regulates the histone H4 acetylation levels at lysine 5, 8, and 16, suggesting that the *O*-GlcNAcylation of NSL3 at Thr755 is required for maintaining the integrity and holoenzyme activity of the MOF/NSL complex. In colony formation assays, we found that the integrity of the complex impacts the proliferation of the lung carcinoma type II epithelium-like A549 cells. Taken together, our results provide new insight into the elucidation of the molecular mechanism of the MOF/NSL complex.

## 1. Introduction

The dynamic and reversible intracellular *O*-GlcNAcylation is controlled by the OGTand glycoside hydrolase *O*-GlcNAcase (OGA) [1]. Among them, OGT is responsible for adding the sugar nucleotide UDP-GlcNAc (*O*-GlcNAc) groups to Serine (Ser)/Threonine (Thr) residues of cytosolic and nuclear proteins [2], whereas OGA catalyzes the reverse reaction to remove the *O*-GlcNAc groups from the substrate proteins [3]. In cells, *O*-GlcNAcylation as a nutrient sensor has been involved in fundamental cellular processes including gene transcription [4], cell cycle progression [5], apoptosis [6], and cell signaling [7]. Once the balance of intracellular *O*-GlcNAcylation levels is broken, it will lead to many diseases such as diabetes, cardiovascular diseases, cancer, and neurodegenerative diseases [2,8].

Increasing evidence has shown that functional responses of the target nucleocytoplasmic and mitochondrial proteins are caused by *O*-GlcNAcylation [9,10]. Crosstalk between *O*-GlcNAcylation and its downstream proteins may provide executive instructions or recognition platforms for subsequent recruitment of proteins to start the specific biological processes [11,12]. For example, OGT directly binds to, and *O*-GlcNAc-modifies, Ser 549 in C-terminus of SIRT1 (Sirtuin 1) under oxidative, genotoxic, and metabolic stress stimuli [13], thereby regulating metabolism, genome stability, and stress responses [14,15]. Also, *O*-GlcNAc-modified SIRT1 enhanced its deacetylase activity, and promoted cell survival [13]. What is more, *O*-GlcNAcylation of host cell factor 1 (HCF-1) on proteolysis repeat domain at the Thr11 site can provide instructions for HCF-1 proteolysis [16].

In addition to free form, OGT in cells could be assembled in a variety of protein complexes to coordinate intracellular biological processes. For instance, OGT/HCF-1 complex modulates the gluconeogenesis pathway through stabilizing the master regulator of gluconeogenesis, PGC-1 α [17]. Recent studies have found that OGT could form complexes with TET (ten-eleven translocation) family proteins including TET1, TET2, and TET3 which mainly catalyze the conversion of 5-methylcytosine (mC) to 5-hydroxymethylation (hmC) [18] to initiate specific functions. OGT binds to and stabilizes TET1, and thereby increases chromatin accessibility [18]; while the complex of OGT and TET2/3 can recruit the SET1/COMPASS complex via *O*-GlcNAcylatinon of HCF-1, a subunit of the SET1/COMPASS complex, therefore promoting the catalytic activity of H3K4 tri-methylation (H3K4me3) by SET1/COMPASS [19]. Moreover, as we reported before, OGT isoform 1 was involved in the formation of the human MOF-containing NSL histone acetyltransferase (HAT) complex [20]. Our data showed that NSL3, a specific subunit of the NSL complex, can be stabilized by OGT1-mediated *O*-GlcNAcylation. This modification further enhanced the enzyme activity of the NSL complex, thereby facilitating the global acetylation level of histone H4 in cells [11]. 

The ubiquitin proteasome pathway, conserved from yeast to human, is required for the targeted degradation of most proteins in cells [21]. Traditional ubiquitin modification is an ATP-dependent process carried out by three classes of enzymes including ubiquitin activating enzyme (E1), ubiquitin conjugating enzyme (E2), and ubiquitin ligase (E3) [22,23]. However, sometimes this process is not strictly followed. The research results from the Scaglione KM group demonstrate that the ubiquitin conjugating enzyme UBE2W attaches ubiquitin to the N-terminus of ataxin-3 and directs ubiquitin-degradation of ataxin-3 [24], suggesting that ubiquitin E2 enzyme may also directly bind to the substrate and promote its degradation. It is worth noting that OGT-mediated *O*-GlcNAc-modification is closely related to the degradation pathway of certain proteins. For instance, genome-wide analysis has found that *O*-GlcNAcylation of H2BS112 frequently co-localizes with H2BK120 mono-ubiquitination [25], suggesting the crosstalk between two modifications. In line with this, the stability and proteasome-degradation of MLL5 (Mixed Lineage Leukemia 5) protein are cooperatively regulated by OGT/*O*-GlcNAcylation and ubiquitin-specific protease 7 (USP7) [26]. Furthermore, OGT-mediated *O*-GlcNAcylation of EZH2 (Enhancer of zeste homolog 2) at S75 not only stabilizes EZH2, but also facilitates H3K27 tri-methylation [27]. 

We previously reported that OGT1 regulated global histone H4 acetylation through stabilizing NSL3. However, there are still many issues that need to be further clarified, such as (i) which region and specific amino acid site of NSL3 are *O*-GlcNAc-modified by OGT1? (ii) Does the *O*-GlcNAcylation of NSL3 at specific sites affect the NSL3 stability? If so, whether this stability is related to the ubiquitin-proteasome pathway of NSL3? (iii) How does the *O*-GlcNAcylation of NSL3 at specific sites impact the integrity and the holoenzyme activity of NSL complex? In an effort to resolve the above questions, a series of biochemical and molecular biological experimental approaches were used in this paper. As we describe below, our results demonstrate a new mechanism, showing that *O*-GlcNAc-modification of NSL3 at Thr755 site by OGT1 is required for maintaining the holoenzyme activity of the MOF/NSL HAT complex.

## 2. Results

### 2.1. OGT1 Stabilized NSL3 through O-GlcNAcylating Its C-Terminus

**Figure 1:**
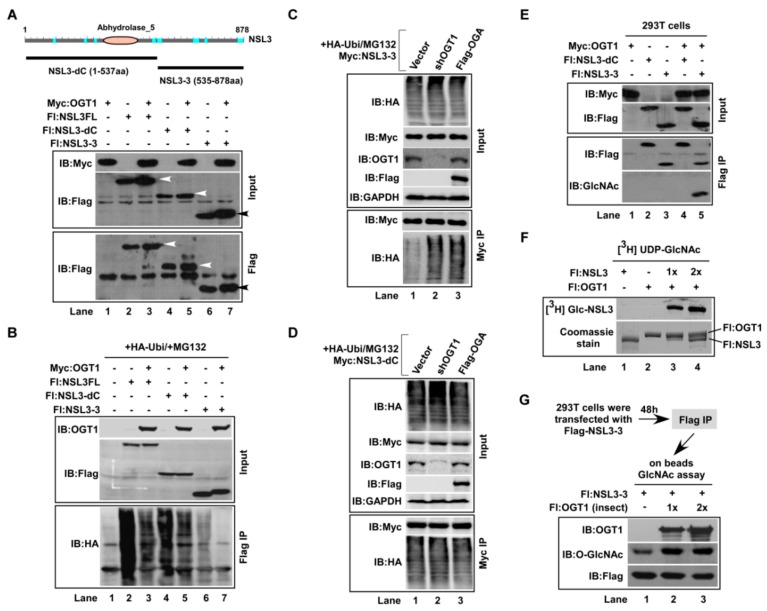
*O*-linked N-acetylglucosamine (*O*-GlcNAc) transferase (OGT1) stabilized nonspecific lethal protein 3 (NSL3) through *O*-GlcNAcylating its C-terminus. “+, −” in figures caption means with or without. (**A**) Stabilization of NSL3 by OGT1: full-length or deletion mutants of NSL3 (upper panel) were co-transfected with OGT1 in 293T cells, and expressed proteins were analyzed using Western blot (WB) with anti-Flag or anti-Myc antibodies (lower panel). (**B**) Declined ubiquitin-mediated degradation of NSL3 by OGT1: degradation of NSL3FL or deletion mutants of NSL3 by OGT1 was observed in the presence of HA-ubiquitin and MG132 in 293T cells. (**C**,**D**) Regulation of *O*-GlcNAcylation on ubiquitin-mediated degradation of NSL3: ubiquitin-mediated NSL3-3 and NSL3-dC degradation was examined in transient transfection of shOGT1 or OGA 293T cells in the presence of MG132. (**E**) *O*-GlcNAc modification of NSL3-3 C-terminus by OGT1: 293T cells were co-transfected with Myc-OGT1 and Flag-tagged NSL3-dC or NSL3-3, and the *O*-GlcNAc modification on NSL3 was then detected by WB with anti-*O*-GlcNAc antibody followed by Flag IP at 48 h after transfection. (**F**) In vitro *O*-GlcNAc transferase assay: insect cells expressed and purified OGT1 and NSL3 proteins were incubated with UDP-[^3^H] GlcNAc in the presence of reaction buffer. *O*-GlcNAc modification on recombinant NSL3 was measured by autoradiography (upper), recombinant OGT1 and NSL3 were visualized by Coomassie Brilliant Blue staining (lower). (**G**) On beads *O*-GlcNAc-assay: 293T cells were co-transfected with Flag-NSL3-3 and OGT1, 48 h after transfection, bound Flag-NSL3-3 was subjected to in vitro *O*-GlcNAc transferase assay. Modified NSL3-3 was detected with specific anti-*O*-GlcNAc antibody.

We previously demonstrated the regulation of OGT1 on NSL3 stability through *O*-GlcNAc-modifying NSL3 [11]. However, it is unclear which amino acid residue of NSL3 can be *O*-GlcNAcylated by OGT1 and how *O*-GlcNAcylation of this site affects the NSL3 stability. Thus, in order to clarify the region in which NSL3 can be *O*-GlcNAc-modified, the deletion mutants were first constructed (Figure 1A, upper). Like full length NSL3 (NSL3FL), both NSL3-dC and NSL3-3 deletion mutants were also stabilized by co-transfecting with OGT1 (Figure 1A, lower, lanes 3, 5 and 7). Next, to know whether the role of OGT1 stabilizing NSL3 is connected to its inhibition of the ubiquitin-proteasome pathway, co-transfection/co-immunoprecipitation (co-IP) experiments were carried out in the presence of HA-ubiquitin and MG132 (a proteasome inhibitor). The levels of NSL3 ubiquitination were then measured by Western blot (WB) with anti-HA antibody after Flag IP. As shown in Figure 1B, compared with the NSL3-alone group (lanes 2, 4, 6), the ubiquitination of full-length and deletion mutant NSL3-3 were significantly decreased by co-transfecting with OGT1 (Flag IP, lanes 3 and 7), while the changes of NSL3-dC were not remarkable (lane 5). In contrast, the ubiquitination of NSL3-3 was reversed quite obviously by knocking down OGT1 or overexpressing an *O*-GlcNAc hydrolase OGA (Figure 1C, lane 2–3 compared to lane 1). Again, no obvious ubiquitination changes of NSL3-dC were visible (Figure 1D). The above results suggest the possibility of *O*-GlcNAcylation at C-terminal NSL3. This speculation was confirmed by the next experiment. As shown in Figure 1E, Flag IP was performed in Flag NSL3-dC/OGT1 or Flag-NSL3-3/OGT1 co-transfected 293T cells. *O*-GlcNAcylation on NSL3 was then measured using anti-*O*-GlcNAc antibody. As a result, *O*-GlcNAc modification was only detected by WB on Flag-NSL3-3 (lane 5). To further verify this result, using established in vitro *O*-GlcNAc-transferease assay (Figure 1F, lanes 3–4), on beads *O*-GlcNAc-transferase assay was performed. As shown in Figure 1G, *O*-GlcNAc-modification on NSL3-3 was determined by WB using anti-*O*-GlcNAc antibody (lanes 2–3). 

### 2.2. Ubiquitin-Conjugating Enzyme UBE2S, But Not UBE2N, Directly Bound to NSL3

**Figure 2:**
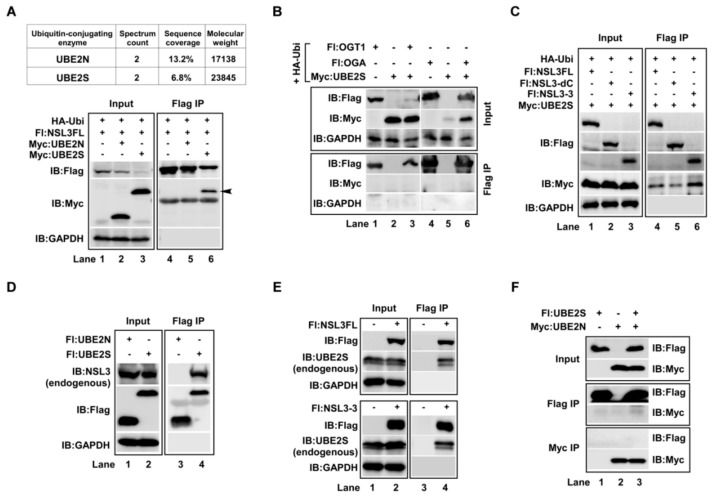
Ubiquitin-conjugating enzyme UBE2S, but not UBE2N, directly bound to NSL3. “+, −” in figures caption means with or without. (**A**) NSL3FL bound to UBE2S. UBE2N and UBE2S were identified by mass spectrometry in anti-Flag eluate from stably expressing Flag-NSL3 293FRT cells (upper). 293T cells were co-transfected with Flag-NSL3FL and Myc-UBE2N or Myc-UBE2S, and the interaction between proteins was measured by WB with anti-Flag or anti-Myc antibodies followed by Flag IP (lower). (**B**) No-interaction between UBE2S and OGT1 or OGA. Co-transfection and co-IP were performed as designed. (**C**) UBE2S and NSL3FL were widely combined with each other. Myc-UBE2S was co-transfected with Flag-tagged NSL3FL or deletion mutants of NSL3. Forty-eight hours after transfection, the interaction between UBE2S and NSL3 was visualized by WB. (**D**) Flag-UBE2S bound to endogenous NSL3. 293T cells were transfected with Flag-tagged UBE2N or UBE2S, and Flag-IP was performed after 48 h transfection. Bound endogenous NSL3 was detected using WB with anti-NSL3 specific antibody. (**E**) Flag-NSL3FL (upper) and Flag-NSL3-3 (lower) bound to endogenous UBE2S. (**F**) No-interaction between UBE2S and UBE2N.

Two ubiquitin-conjugating enzymes, UBE2N and UBE2S, were identified by protein mass spectrometry from the Flag IP eluate of Flag-NSL3 stably expressing cells. The spectrum count and sequence coverage were shown in Figure 2A, upper. To investigate the interaction between NSL3 and UBE2N/UBE2S, Myc- or Flag- tagged UBE2N and UBE2S plasmids were constructed. Then, co-transfection/co-IP experiments were designed as shown in Figure 2A, lower. The result indicated that the NSL3 and UBE2S, but not UBE2N, bound each other (lane 6). In view of the combination of OGT1 and NSL3, the possibility of UBE2S interacting with OGT1 or OGA was then examined (Figure 2B). As a result, UBE2S was neither combined with OGT1 nor with OGA (Flag IP/IB:Myc). To further map the binding region of NSL3 with UBE2S, co-IP experiments were carried out using NSL3FL and its deletion mutants. As shown in Figure 2C, UBE2S was widely combined with NSL3 (Flag IP/IB:Myc, lane 4–6), however, the affinity with NSL3-3 was much higher than the NSL3FL and NSL3-dC. In order to confirm the reliability of this result, it was examined whether the endogenous NSL3 protein could be precipitated by exogenous Flag-UBE2S. As expected, endogenous NSL3 was immunoprecipitated by transient transfected Flag-UBE2S, but not Flag-UBE2N (Figure 2D, Flag IP/IB:NSL3). Furthermore, endogenous UBE2S was immunoprecipitated by exogenous Flag-NSL3FL and Flag-NSL3-3 (Figure 2E, lane 4). Finally, to further rule out the possibility of combining UBE2S and UBE2N, co-transfection/co-IP experiments were carried out. However, there was no evidence to prove the interaction between UBE2S and UBE2N (Figure 2F). In summary, the above results strongly suggest that ubiquitin-conjugating enzyme UBE2S, but not UBE2N, directly bound to NSL3 and may be involved in the regulation of the stability of NSL3. Thus, in the following studies, we focused our sight on the coordinative regulation of UBE2S and OGT1 in NSL3 stability. 

### 2.3. O-GlcNAcylation of NSL3 by OGT1 Was Tightly Associated with UBE2S-Mediated Ubiquitin-Degradation Pathway

**Figure 3:**
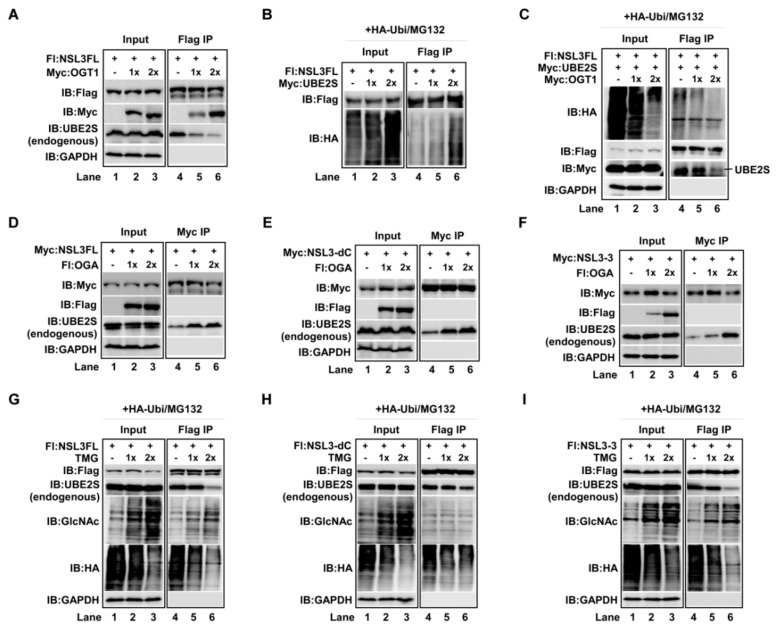
*O*-GlcNAcylation of NSL3 by OGT1 was tightly associated with UBE2S-mediated ubiquitin-degradation pathway. “+, −” in figures caption means with or without. (**A**) Dose-dependent inhibition of OGT1 on the interaction between endogenous UBE2S and NSL3: increasing amounts of Myc-OGT1 were co-transfected with Flag-NSL3 in 293T cells. Forty-eight hours after transfection, anti-Flag eluates were analyzed by WB with anti-UBE2S antibody. (**B**) Increased ubiquitin-degradation levels of NSL3 in the presence of UBE2S: degradation status of exogenous NSL3 was tested after Flag IP using anti-HA antibody. (**C**) Suppressed UBE2S-mediated degradation of NSL3 by OGT1: UBE2S mediated degradation of NSL3 was measured in Myc-OGT1 overexpressing cells. Simultaneously, the interaction between NSL3 and UBE2S was detected with anti-Myc antibody after Flag IP. (**D**–**F**) Dose-dependent increase of endogenous UBE2S bound to NSL3 by co-transfecting Flag-OGA with NSL3FL and its deletion mutants. (**G**–**I**) UBE2S-mediated degradation status of NSL3FL and its deletion mutants by TMG: 293T cells co-transfected with Flag-NSL3FL, NSL3-dC or NSL3-3 and HA-ubiquitin were treated with 0, 1, and 2 μM TMG for 4h, then ubiquitin-mediated degradation of NSL3 was estimated by WB with anti-HA antibody and the *O*-GlcNAcylation level of NSL3 was estimated by WB with anti-*O*-GlcNAc antibody after Flag IP. The endogenous UBE2S protein levels were analyzed using WB with anti-UBE2S antibody.

Given that NSL3 binds to both OGT1 and UBE2S, it is not difficult to imagine that OGT1 and UBE2S may jointly regulate the stability of NSL3 protein. To address this issue, the competitive binding experiments were designed as shown in Figure 3A. The results revealed that the binding activities between NSL3 and endogenous UBE2S were dose-dependently decreased by increasing the amount of OGT1 (Flag IP, IB:UBE2S), suggesting the interaction among those three proteins. Next, to understand whether UBE2S-mediated ubiquitin pathway affects the role of OGT1 in stabilizing NSL3, the effects of UBE2S on NSL3 protein degradation were measured under presence of HA-ubiquitin and MG132. As shown in Figure 3B, dose-dependent increase of ubiquitin-degradation of NSL3 was verified by co-transfecting NSL3 with increasing amount of UBE2S (Flag IP/IB:HA, lanes 5–6 compared to lane 4). However, this function of UBE2S on NSL3 was reversed by adding OGT1 (Figure 3C, lanes 2–3 and 5–6, compared to lanes 1 and 4). In addition, the binding affinity between NSL3 and UBE2S was dose-dependently decreased (Flag IP, IB: Myc). This result suggests that *O*-GlcNAcylation of NSL3 by OGT1 inhibits the binding of UBE2S to NSL3, thereby enhancing the stability of NSL3 protein. The subsequent experimental results also support this view. Contrary to OGT1, OGA remarkably increased the binding affinity between NSL3 and endogenous UBE2S (Figure 3D–F, Myc IP/IB:UBE2S, lanes 5–6 compared to lane 4). Furthermore, OGA specific inhibitor thiamet-G (TMG) was used in Figure 3G to 3I experiments. As expected, the *O*-GlcNAc-modification levels on exogenous Flag-NSL3FL (Figure 3G, lanes 4–6, IB:GlcNAc) and Flag-NSL3-3 (Figure 3I, lanes 4–6, IB:GlcNAc) were visibly increased. This further resulted in decreased ubiquitin-mediated degradation. However, the *O*-GlcNAcylation level and ubiquitin-mediated degradation of Flag-NSL3-dC was not changed by treating cells with TMG (Figure 3H, lanes 4–6, IB:GlcNAc), supporting our earlier result that the *O*-GlcNAcylation occurs in the C-terminal region of NSL3. In addition, the endogenous UBE2S protein levels were dose-dependently decreased by increasing amount of TMG (IB:UBE2S).

### 2.4. O-GlcNAc-Modification of NSL3 by OGT1 Mainly Occurred at Thr755

**Figure 4:**
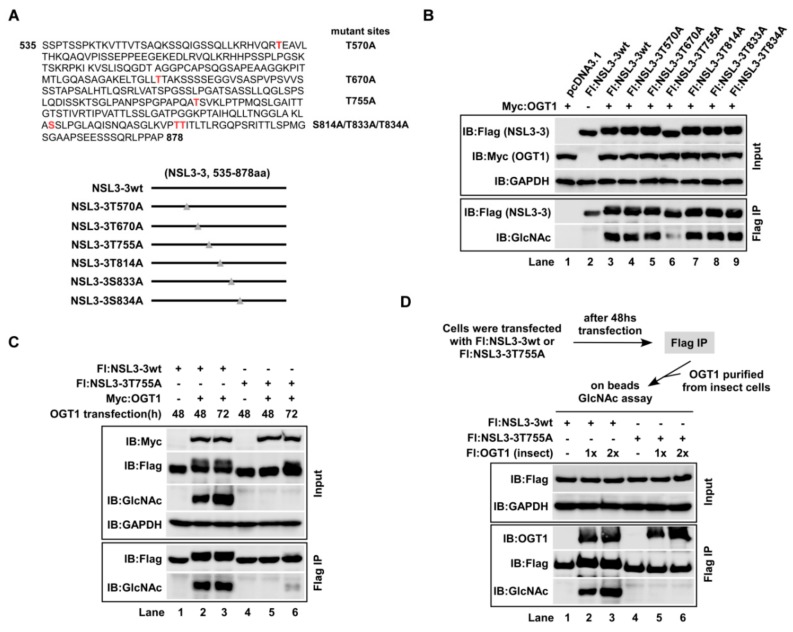
*O*-GlcNAc-modification of NSL3 by OGT1 mainly occurred at Thr755. “+, −” in figures caption means with or without. (**A**) Potential *O*-GlcNAc modification site at the C-terminus of NSL3: 293T cells were co-transfected with Flag-NSL3-3 and Myc-OGT1, potential *O*-GlcNAcylation sites were identified by mass spectrometry after Flag IP (upper). Flag-tagged point mutation plasmids were constructed for all potential *O*-GlcNAcylation sites. (**B**) Expression of recombinant proteins and their modification by OGT1: 293T cells were transfected with indicated point mutants of NSL3-3 for 48 h, then the recombinant protein expression levels were measured by WB with anti-Flag antibody. The *O*-GlcNAc modification site on NSL3-3 was detected by anti-*O*-GlcNAc antibody followed by Flag IP. (**C**) Confirmation of the major *O*-GlcNAcylation site on NSL3 C-terminus: Flag-tagged wild type or T755A point mutant of NSL3-3 was co-transfected with OGT1 into 293T cells for 48 h or 72 h, and the protein expression levels and the *O*-GlcNAcylation status were analyzed by WB with anti-Flag and anti-*O*-GlcNAc antibodies. (**D**) On beads *O*-GlcNAc assay: over-expressed Flag-NSL3-3wt or NSL3-3 T755A point mutant was immunopurified using anti-Flag M2 agarose, after washing, on beads in vitro *O*-GlcNAc assay was performed. Modified *O*-GlcNAcylation was visualized by WB with anti-*O*-GlcNAc antibody.

According to the former data we obtained, the *O*-GlcNAc modification may occur at the C-terminal region of NSL3. To further identify the putative *O*-GlcNAcylation sites of NSL3, Flag-tagged NSL3-3 was transiently co-transfected with OGT1 into 293T cells for 48 h, and anti-Flag eluate was then analyzed via mass spectrometry. As shown in Figure 4A, multiple potential *O*-GlcNAc modification sites on NSL3-3 including Thr570, Thr670, Thr755, Ser814, Thr833, and Thr834 were presumed. A point mutation plasmid with a Flag-tag was constructed for each site. Then, wild type or point mutants of NSL3-3 were co-transfected with OGT1 into 293T cells for 48 h. *O*-GlcNAc-modification on NSL3-3 was detected with WB using anti-*O*-GlcNAc antibody after Flag IP. In line with previous results, the protein size of *O*-GlcNAc-modified NSL3-3wt by OGT1 was higher than non-modified NSL3-3 on SDS-PAGE gel (Figure 4B, lane 3 compared to lane 2). It is worth noting that all mutants except for NSL3-3 T775A can be *O*-GlcNAc-modified by exogenous OGT1, suggesting that the T775 site of NSL3 may be the major *O*-GlcNAc-modifying site. To further confirm this hypothesis, cells were harvested at 48 h and 72 h after co-transfection of OGT1 and wild type NSL3-3 (NSL3-3wt) or mutant NSL3-3 (NSL3-3mt), and the *O*-GlcNAc-modified proteins were measured with WB using anti-*O*-GlcNAc antibody. As shown in Figure 4C, NSL3-3wt can be *O*-GlcNAc-modified by OGT1 as usual, whether it was 48 h or 72 h (lanes 2–3 compared to lane 1). However, no or weak *O*-GlcNAc- signal was seen on the NSL3-3 T775A mutant (lanes 5–6). Furthermore, on beads in vitro *O*-GlcNAc-transferase assay was carried out to clarify the above results. As expected, anti-Flag immunopurified exogenous NSL3-3wt, but not T755A mutant, was also *O*-GlcNAc-modified by recombinant OGT1 (Figure 4D, Flag IP, IB:GlcNAc).

### 2.5. O-GlcNAcylation of NSL3 Thr755 Site by OGT1 Was Connected to the NSL3 Stability

**Figure 5:**
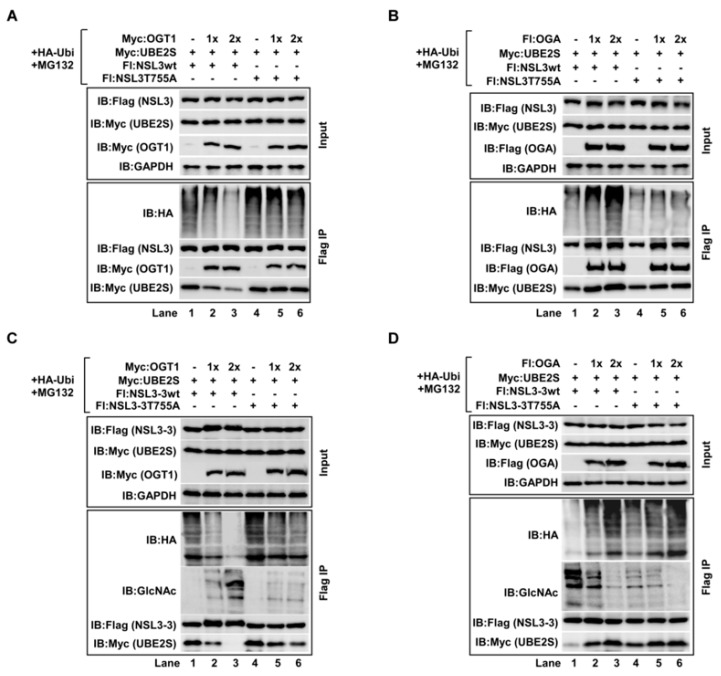
Stabilization of NSL3 was regulated by *O*-GlcNAcylation of NSL3 Thr755 site. “+, −” in figures caption means with or without. (**A**,**C**) *O*-GlcNAcylation of Thr755 site mediated NSL3 stability. Increasing amounts of OGT1 were co-transfected with full length of NSL3wt (NSL3-3wt) or NSL3T755A (NSL3-3 T755A) into 293T cells for 48 h in the presence of HA-ubiquitin, UBE2S, and MG132. Then, the protein degradation, interaction between NSL3 and OGT1 or NSL3 and UBE2S were analyzed by Western blot with indicated antibodies. (**B**,**D**) A similar experiment was performed using OGA and NSL3wt (NSL3-3wt) or NSL3T755A (NSL3-3 T755A).

We have mentioned that *O*-GlcNAcylation of NSL3 by OGT1 regulated its ubiquitin-degradation mediated protein stability. Therefore, the determination of the NSL3 *O*-GlcNAcylation site prompted us to further elucidate whether the stabilization of NSL3 by OGT1 is connected with this site. In order to confirm this hypothesis, full length wild type or T755A mutant of NSL3 was co-transfected with increasing amounst of OGT1 in the presence of UBE2S, HA-ubiquitin, and MG132. As shown in Figure 5A, in agreement with the data before, OGT1 not only suppressed the binding activity between UBE2S and NSL3, but also decreased the ubiquitination level of NSL3wt in a dose-dependent manner (Flag IP, lanes 2–3). However, this phenomenon was disappeared by co-transfecting OGT1 with NSL3 T755A mutant (Flag IP, lanes 5–6, compared to lane 4). Furthermore, the opposite results were obtained by co-transfecting OGA with NSL3wt or NSL3 T755A mutant. Greater ubiquitin modification on NSL3 and dose-dependent increase of UBE2S were seen in co-transfection of OGA and NSL3wt (Figure 5B, Flag IP, lanes 2–3). Conversely, the role of OGA on NSL3 T755A mutant was remarkably weakened, indicating that OGT1 regulated the ubiquitin-degradation pathway-mediated function of NSL3 through the *O*-GlcNAc-modification at NSL3 Thr755 site. Next, a similar experiment was performed using the NSL3-3 truncation mutant to further confirm our conclusion. As expected, co-transfection of NSL3-3wt with OGT1 simultaneously inhibited the binding ability between NSL3-3 and UBE2S, and the ubiquitin degradation levels of NSL3-3 (Figure 5C, Flag IP, lanes 2–3). When Thr755 site on NSL3-3 was mutated, the ubiquitin-degradation of NSL3-3 T755A was significantly inhibited (Flag IP, lanes 5–6). These results were further clarified by co-transfection of OGA with NSL3-3wt or NSL3-3 T755A mutant (Figure 5D). Based on the above results, it suggests that the regulation of OGT1 to NSL3 stability is tightly associated with its modulating ubiquitin-degradation pathway through *O*-GlcNAcylating Thr755 site of NSL3.

### 2.6. O-GlcNAcylation of NSL3 at Thr755 Site Was Required for Maintaining the Integrity of NSL Complex and Its Holoenzyme Activity

**Figure 6:**
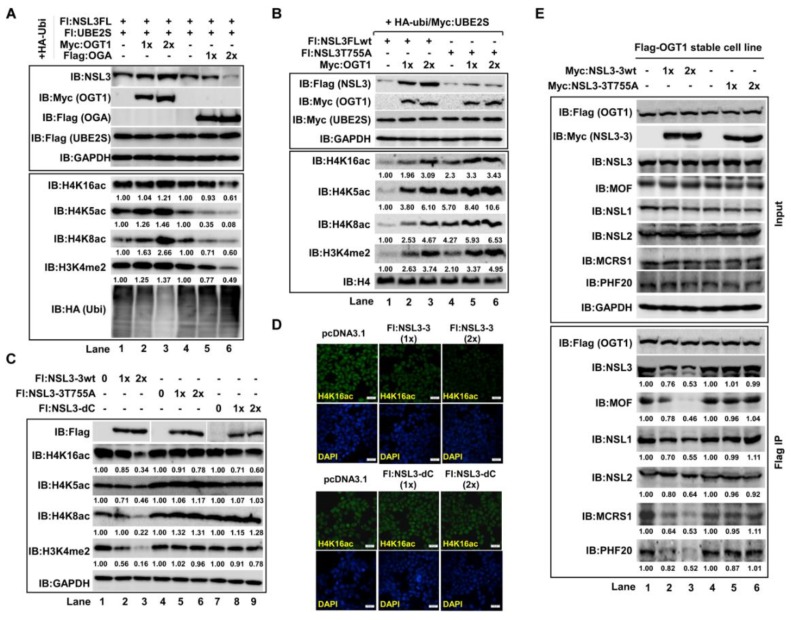
*O*-GlcNAc modification at NSL3 Thr755 site was required for maintaining the integrity of NSL complex and its holoenzyme activity. “+, −” in figures caption means with or without. (**A**) OGT1/*O*-GlcNAcylation mediated changes in global histone H4 acetylation and H3K4me2: increasing amounts of OGT1 or OGA were co-transfected with NSL3 into 293T cells in the presence of UBE2S and HA-ubiquitin. Global histone H4 acetylation at lysine K5, K8, and K16 and H3K4me2 levels was detected with corresponding antibodies. Ubiquitin-mediated NSL3 degradation was visualized with anti-HA antibody. (**B**–**C**) Effects of NSL3T755 site *O*-GlcNAcylation on holoenzyme activity: increasing amounts of OGT1 were co-transfected with NSL3wt or NSL3T755A into 293T cells in the presence of UBE2S and HA-ubiquitin. Modified histones were visualized by WB with specific acetylated or methylated antibodies (**B**). Further verification experiments were performed by transient transfecting Flag-tagged NSL3-3wt, NSL3-3T755A, and NSL3-dC plasmids. Global histone modification changes were measured with specific acetylated or methylated antibodies (**C**). (**D**) Cells were transfected with NSL3-3wt and NSL3-3 deletion (NSL3-dC) mutants. Forty-eight hours after transfection, immunofluorescence staining was carried out to detect the global histone H4K16ac status. (**E**) Effects of NSL3 Thr755 site *O*-GlcNAcylation on the integrity of NSL complex: the stably overexpressing Flag-OGT1 cells were transfected with gradient doses of NSL3-3wt and NSL3-3 T755A; Anti-Flag- agarose eluates were subjected to SDS-PAGE, and the interaction were detected by WB with indicated antibodies. The numbers in A, B, C, and E reveal the quantified protein levels by ImageJ. Normalization was done by dividing the target signal to GAPDH signal.

Considering that both OGT1 and NSL3 are components of the NSL complex, we speculate that the stability of the NSL3 protein by OGT1 may be related to maintaining the structural integrity of the complex. As expected, global histone H4 acetylation at lysine K5, K8, and K16 and H3K4me2 levels were dose-dependently increased by stabilizing NSL3 through co-transfection of OGT1 and NSL3 (Figure 6A, lanes 2–3 compared to lane 1). Contrary to OGT1, OGA decreased those in co-transfection with NSL3 in cells (Figure 6A, lanes 5–6 compared to lane 4), suggesting that the *O*-GlcNAcylation mediated NSL3 stability directly impacts the holoenzyme activity of NSL complex. In order to further confirm this conclusion, the NSL3 T755A and NSL3 C-terminal deletion (NSL3-dC) mutants were used in the next experiments. Compared to the NSL3wt groups, much higher levels of histone H4 acetylation and H3K4me2 were observed in co-transfection of NSL3 T755A and OGT1 groups (Figure 6B, lanes 4–6 compared to lanes 1–3). The possible reason is because there are more OGT1 involved in *O*-GlcNAc-modifying endogenous NSL3, resulting in more stabilized NSL complex. The same reason can explain why the levels of acetylation of H4 and H3K4me2 are reduced after overexpression of NSL3-3 (Figure 6C, lanes 1–3, Figure 6D, upper panel). As we mentioned before, NSL3 was *O*-GlcNAcylated at Thr755 site on C-terminus, thus a large amount of endogenous OGT1 was consumed in *O*-GlcNAcylation of exogenous NSL3-3, resulting in a decrease in endogenous NSL complex. Therefore, the integrity of the endogenous NSL complex was not affected by overexpressing the Flag-NSL3-3 T755A and C-terminal deletion mutant (Flag-NSL3-dC) and the level of histone H4 acetylation and H3K4me2 in cells did not fluctuate greatly (Figure 6C, lanes 4–9; Figure 6D, lower panel). We then endeavored to extend our findings to the influence of NSL3 *O*-GlcNAcylation on the integrity of NSL complex. To do that, the stably overexpressing Flag-OGT1 cells were established. Then, we competitively inhibited the level of *O*-GlcNAcylation of endogenous NSL3 by transient transfecting NSL3-3, and the changes in NSL complex components were analyzed by WB with corresponding specific antibodies following anti-Flag IP. As shown in Figure 6E, the subunits of NSL complex, including NSL3, MOF, MCRS1, and PHF20, were significantly decreased in a dose-dependent manner while NSL1 and NSL2 were also reduced slightly. However, this phenomenon was not seen in cells that overexpressed Flag-NSL3-3 T755A mutants. Taken together, the *O*-GlcNAc-modification of NSL3 at Thr755 site by OGT1 not only stabilized the NSL3 protein, but more importantly, directly affected the structural integrity and holoenzyme activity of the NSL complex. 

**Figure 7:**
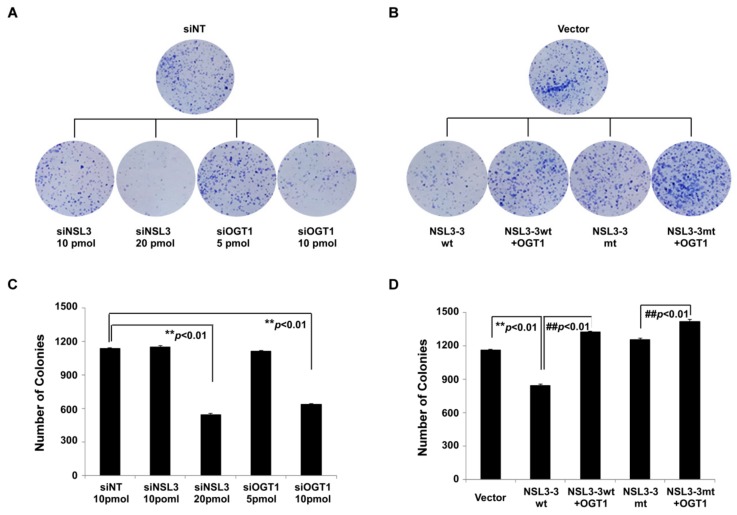
The OGT1/*O*-GlcNAcylation-mediated NSL3 stability impacted the proliferation of A549 cells. (**A**,**B**) Colony formation assay (*n* = 3): A549 cells were treated with indicated siRNA (**A**) or transfected with plasmids (**B**) as indicated design. Forty-eight hours later, cells were collected and split into a new 6-well plate with 2400 cells/well. Two weeks later, formed colonies were stained with Giemsa. (**C**,**D**) Number of colonies: images of stained colonies containing >20 cells were scored as positive. The number of foci >100 μm was counted. Counted numbers of colonies are shown as a bar graph; ** *p* < 0.01 (*t*-test), compared with siNT or vector group; ^##^ *p* < 0.01 (*t*-test), compared with NSL3-3wt or NSL3-3mt only group.

To gain the insight into the role of OGT1/O-GlcNAcylation mediated integrity of NSL complex on the biological function of cells, colony formation of A549 cells as a preliminary study was carried out. As shown in Figure 7A, knocking down NSL3 or OGT1 caused a significant reduction in the number of clones formed. The number of clones was quantified into a bar graph as shown in Figure 7C. Statistically significant differences (** *p*< 0.01) were found in 20 pmol siNSL3 and 10 pmol siOGT1 groups compared to siNT group, suggesting that the integrity of NSL complex is required for cell proliferation of A549. Next, in order to further confirm the role of the integrity of NSL complex in regulating cell proliferation, colony formation assays were performed after transfection of wild type or T755A point mutant NSL3-3 in the presence or absence of OGT1 (Figure 7B). As a result, compared to the vector-control group, NSL3-3wt only decreased colonies formed (Figure 7D, column 2, ** *p* < 0.01), however, the number of colonies was increased by co-transfecting NSL3-3wt and OGT1 (column3, ^##^ *p* < 0.01 compared to NSL3-3wt group), suggesting that exogenous NSL3-3 consumes a large amount of endogenous OGT1 to O-GlcNAc-modify NSL3-3, therefore producing results similar to knockdown of NSL3. As expected, there was no significant change in the number of colonies between the NSL3-3mt transfected- and vector- control groups (column 4). However, increased colony numbers were seen in co-transfection of NSL3-3mt and OGT1 (column 5, ^##^ *p* < 0.01 compared to NSL3-3mt group), suggesting that the O-GlcNAc modification and stabilization of NSL3 by OGT1 may have impacted the proliferation of A549 cells.

## 3. Discussion

Research data over recent years has revealed that *O*-GlcNAc-modification of specific substrate cellular proteins on Ser/Thr residues mediated significant intracellular biological functions of downstream proteins. As one of the post-translational modifications, the extensive crosstalk between *O*-GlcNAcylation and other epigenetic modifications including but not limited to phosphorylation, acetylation, and ubiquitination came into view [26,27]. In this paper, using a series of biochemical experiments, we demonstrated that OGT1 maintained the stabilization of NSL3 via adding *O*-GlcNAc group to Thr755 site on the C-terminal NSL3. This modification suppressed the binding activity between NSL3 and UBE2S, thereby inhibiting the ubiquitin-proteasome degradation of NSL3. 

It is well known that OGT1-mediated *O*-GlcNAc-modification is closely related to protein stability. For example, tumor suppressor p53 can be stabilized and activated by *O*-GlcNAcylation at Ser149 residue in response to stress [8]. We previously reported that OGT1 directly binds to and stabilizes NSL3, a specific subunit of the NSL complex through *O*-GlcNAc-modifying NSL3 [11]. However, the specific *O*-GlcNAc modification site on NSL3 by OGT1 and its precise mechanism for stabilizing NSL3 remains unclear. Based on in vivo and in vitro *O*-GlcNAc transferase assays, we defined that the C-terminus of NSL3 can be *O*-GlcNAc-modified by OGT1. Analysis of mass spectrometry combined with a series of biochemical and molecular biology experiments, Thr755 residue on NSL3 C-terminus was speculated as a major *O*-GlcNAc-modifying site by OGT1. Since this site was mutated, NSL3 can no longer be *O*-GlcNAc-modified by OGT1 (Figure 4C,D). In view of the fact that OGT1 can regulate the stability of NSL3, the role of *O*-GlcNAcylation on Thr755 site in regulating the stabilization of NSL3 was speculated. As expected, compared to the wild type NSL3 group, overexpressing point mutant NSL3 T755A significantly antagonized the role of OGT1 in stabilizing NSL3 in cells (Figure 5A,C), suggesting the importance of Thr755 site on NSL3 in the regulation of OGT1 on the NSL3 stability.

Although the exact mechanism between OGT-mediated *O*-GlcNAcylation and the ubiquitin-degradation pathway remains to be elucidated, the *O*-GlcNAcylation was greatly involved in the protein stability and proteasome degradation pathway. For instance, *O*-GlcNAc modification of Ser435 and Thr440 on MLL5 stabilized its protein level through inhibiting its ubiquitin-degradation pathway, thus further affecting the methylation activity of H3K4 by MLL5 [26]. Moreover, *O*-GlcNAcylation mediated SIRT1 stability in cancer cells directly affected the proteasome degradation of oncogenic transcription factor FOXM1 [28]. In our experiments, we presented evidence that OGT1-mediated *O*-GlcNAcylation stabilized NSL3 protein by inhibiting the ubiquitin degradation pathway of NSL3 (Figure 2 and Figure 3). Mass spectrometry analysis from the Flag IP eluate of Flag-NSL3 stably expressing cells identified UBE2S and UBE2N, both of which are ubiquitin-conjugating enzymes. More importantly is that the direct interaction between NSL3 and UBE2S, but not UBE2N, was confirmed using co-immunoprecipitation experiments. Even more, co-transfection of NSL3 and UBE2S dramatically increased the ubiquitination levels of NSL3, indicating the UBE2S-mediated ubiquitin degradation of NSL3. It is worth noting that the interaction between NSL3 and UBE2S was suppressed by OGT1 in a dose-dependent manner. Simultaneously, the level of NSL3 ubiquitin degradation was weakened by addition of OGT1 (Figure 3). These results strongly suggested that OGT1 and UBE2S may coordinate the regulation of the stability of NSL3 protein in cells. It is worth noting that UBE2S seems to bind to NSL3 in a wide range including the C-terminal deletion mutant (NSL3-dC) in which could not detect *O*-GlcNAc modified signals by WB analysis. Also, the degradation of this region of NSL3 was impacted by OGA and TMG, a specific OGA inhibitor. What counts is that TMG treatment caused an obvious increase of *O*-GlcNAcylation level of NSL3FL and NSL3-3, which was accompanied by reduced ubiquitination levels and gradually decreasing interaction with UBE2S, while none of these changes were particularly noticeable in NSL3-dC. These suggest UBE2S mediated ubiquitin degradation pathway was closely related to *O*-GlcNAcylation. Based on the interaction between UBE2S and NSL3-3 reduced by OGT1 and TMG, UBE2S is more likely to bind to NSL3 that is not *O*-GlcNAc modified. Therefore, it is easy to understand the combination of UBE2S and NSL3-dC. Another possibility is that NSL3-dC region may have weak or transient *O*-GlcNAc modifications although the *O*-GlcNAc-signal could not be seen by WB analysis. In addition, it cannot be ruled out that UBE2S as an ubiquitin-conjugating enzyme may interact with other proteins and then bind to NSL3 together. 

In cells, OGT is frequently assembled as a component into protein complexes, and coordinates with other subunits to perform the corresponding biological functions [12]. For example, OGT can be complexed with TET 2/3 proteins which play a key role in DNA demethylation, and further recruit SET1/COMPASS complex by *O*-GlcNAc-modifying the host cell factor 1 (HCF1) (a subunit of the SET1/COMPASS complex). Also, *O*-GlcNAc-modification of HCF1 promotes catalytic activity of H3K4 tri-methylation (H3K4me3) by SET1/COMPASS [19]. What is more, *O*-GlcNAcylation on EZH2 at Ser75 not only stabilizes the PRC2 complex, but also further affects histone H3K27 tri-methylation activity by PRC2 complex [27]. As we reported previously, both NSL3 and OGT1 are components of MOF-containing NSL HAT complex. Knockdown of NSL3 with specific siRNA resulted in reduced global histone H4 acetylation and H3K4me2 in the presence of OGT1. In contrast, increased levels of those in co-transfection of OGT1 with NSL3FL cells were observed, suggesting that the global histone H4 acetylation and H3K4me2 levels were tightly associated with the status of *O*-GlcNAc-modified NSL3. Therefore, it is easy to understand why co-transfection of OGT1 with NSL3 T755A mutant increased the enzyme activity of NSL HAT complex. The reason is that there are more OGT1 involved in glycosylation of endogenous NSL3. Given that the *O*-GlcNAc modification levels of NSL3 affected the enzymatic activity of the NSL complex, it can be speculated that *O*-GlcNAcylation of NSL3 by OGT1 plays a critical role in maintaining the structural stability of the NSL complex. In line with this view, analysis of NSL complex components in Flag IP eluate after co-transfection of OGT1 and NSL3-3wt revealed that endogenous subunits of NSL complex including NSL3, MOF, NSL1, MCRS1, and PHF20 decreased with the increasing amount of NSL3-3wt, however, this phenomenon was not seen in the cells co-transfected OGT1 with NSL3-3T755A mutant (Figure 6), suggesting that the ratio of *O*-GlcNAcylated endogenous NSL3 directly impacted the NSL complex integrity. It is worth noting that the integrity and stability of the NSL complex directly impacted the proliferation of lung cancer cells A549, as the addition of exogenous NSL3-3wt caused a significant decrease in the number of clones of A549 cells in the colony formation assay (Figure 7).

Taken together, our findings have demonstrated a novel mechanism by which the *O*-GlcNAc modification of NSL3 Thr755 caused by OGT1 contributes to the structural stability of MOF-containing NSL3 HAT complex, thus affecting the holoenzyme activity via regulating UBE2S-mediated proteasome degradation of NSL3. Collectively, these results establish a molecular pathway linking *O*-GlcNAcylation of NSL3 to holoenzyme activity of MOF/NSL3 HAT complex.

## 4. Materials and Methods

### 4.1. Antibodies

Anti-Flag (M2) (A2220)- and anti-Myc (M2)-agarose (A7470), anti-Flag M2 (F3165) monoclonal- and anti-H4K16ac (H9164) polyclonal- antibodies, UDP-GlcNAc (U4375), MG132 (Z-Leu-Leu-al) and TMG (SML0244) were obtained from Sigma (St. Louis, MO, USA). Anti-c-Myc (9E10), and anti-*O*-GlcNAc (IgM, sc-59623) monoclonal antibodies, and anti-OGT1 (H300, sc-32921) polyclonal antibody were purchased from Santa Cruz Biotechnology (Dallas, TX, USA). Anti-H4K5ac (07-327), anti-H4K8ac (07-328), and anti-H3K4me2 (07-030) polyclonal antibodies were from Merck Millipore (Darmstadt, Germany). Anti-MOF (A02757) monoclonal antibody was from Boster Biological Technology (Wuhan, China). Anti-UBE2S (A4658) polyclonal antibody was from ABclonal (Wuhan, China). Anti-histone H4 (16047-1-AP) polyclonal- and anti-HA (66006-2-lg) monoclonal- antibodies were obtained from Proteintech Group (Wuhan, China). Anti-NSL3, anti- NSL1, anti-MCRS1, anti-NSL2, anti-PHF20, and anti-GAPDH rabbit polyclonal antibodies were raised against bacterially expressed proteins (Jilin University, Changchun, China).

### 4.2. Cell Culture

HEK293T and stably expressing Flag-OGT1 HEK293FRT cells were cultured in Dulbecco’s modified Eagle’s medium (DMEM; Gibco, Life Technologies, Waltham, MA, USA), and lung carcinoma type II epithelium-like A549 cells were cultured in PRMI 1640 medium (Gibco, Life Technologies, Waltham, MA, USA) supplemented with 10% fetal bovine serum (FBS; KangYuan Biology, Beijing, China) and 1% penicillin-streptomycin mixture (P/S; Thermo Fisher Scientific, Waltham, MA, USA) at 37 °C in the presence of 5% CO_2_.

### 4.3. Plasmids

The coding region of full-length UBE2N (NM_003348.4), UBE2S (NM_014501), OGT1 (NM_181672), NSL3 (NM_001115016) and point mutants NSL3/ T755A, deletion mutants NSL3-3 (aa535–878) and NSL3-dC (aa1–537), and deletion/point mutant NSL3-3/T570A, NSL3-3/T670A, NSL3-3/T755A, NSL3-3/T814A, NSL3-3/S833A and NSL3-3/S834A with Flag or Myc tag were cloned into pcDNA3.1(-). HA-ubiquitin and 3×Flag-OGA plasmids were gifted by Professor Xianlu Zeng from Northeast Normal University (Changchun, China). 

### 4.4. Expression of Recombinant Proteins in Sf21 Insect Cells

N-terminal Flag-tagged human OGT1 and NSL3 were subcloned into pBacPAK8. Recombinant baculoviruses were generated with the BacPAK baculovirus expression system (Clontech). Recombinant Flag-OGT1 from *Sf21* cells were purified essentially as described [11]. 

### 4.5. In Vitro O-GlcNAc Transferase Assay

Insect cell expressed and purified Flag-tagged full-length NSL3 and OGT1, and intracellular overexpressed wild type or T755A point mutant of NSL3-3 were used in in vitro *O*-GlcNAc transferase assays. The assay reaction (25 µl total Volume), including NSL3, OGT1, and reaction buffer containing 50 mM Tris-HCl (pH 7.4), 12.5 mM MgCl_2_, 0.4 μCi of UDP-[^3^H] GlcNAc (0.1 μCi/μL; Amersham Biosciences) or 1μg/μL cold UDP-GlcNAc, 1 mM dithiothreitol (DTT), were incubated at 37 °C with 550 rpm. After 90 min incubation, the reaction was stopped by 4× SDS sample buffer. Modified proteins were detected by Western blot with anti-*O*-GlcNAc antibody. Radiolabeled proteins were visualized by autoradiography. 

### 4.6. Transient Transfection and Immunoprecipitation (IP)

Flag- or Myc- tagged full-length or deletion mutants or point mutants of NSL3, full-length OGT1, OGA, UBE2N, UBE2S, and HA-tagged ubiquitin plasmids were transiently transfected into HEK 293T cells cultured in 6-well plates (5 × 10^5^ cells/well) using polyethyleneimine (PEI) (catalog number 23966, Poly-sciences). However, for the IP or co-IP experiments, 10-cm tissue culture plates were used. Total transfected plasmids in a 6-well plate were 2µg/well and for a 10-cm plate were 10µg. Forty-eighto hours after transfection, cells were lysed in RIPA Lysate buffer (1% NP-40, 150 mM NaCl, 50 mM Tris-HCl, 10% glycerol, 1mM dithiothreitol (DTT) and complete protease inhibitor cocktails. Flag- or Myc-tagged proteins and their associated proteins in whole-cell lysates were purified on anti-Flag (M2)- or anti-Myc-agarose beads, and the immunoprecipitated proteins were eluted with 4 × SDS buffer directly or 0.2 mg/mL Flag or Myc peptides. Bound proteins were analyzed by WB with anti-Flag or anti-Myc antibody.

### 4.7. Immunofluorescence Staining

HEK293T cells, grown to ~30% confluence in 24-well plates containing a coverslip (8D1007, Nest) on each well, were transfected with Flag-NSL3-3 and Flag-NSL3-dC plasmids. Forty-eight hours later, cells were immunostained according to the method previously described [11]. H4K16ac (1:400 dilution) primary antibody and FITC-conjugated secondary antibody (1:300, Santa Cruz sc-2012) were used. Cell nuclei were stained with Vectashield with DAPI (H-1200) (Vector Laboratories, Inc., Burlingame, CA, USA). Fluorescent images were observed with Olympus BX40F Microscope (Olympus Corporation, Miyazaki, Japan) with a silicon immersion 40× objective. 

### 4.8. Colony Formation Experiment

A549 cells, grown to ~30% confluence in 6-well plates, were treated with scramble siRNA, siNSL3, or siOGT for knockdown NSL3 and OGT1, or were transfected with wild type or T755A point mutant of NSL3-3 with or without OGT1 plasmids. Forty-eight hours later, cells were digested with trypsin, re-suspended in RPMI 1640 medium, and split into a new 6-well plate with 2400 cells/well. Two weeks later, the cell were fixed with 4% paraformaldehyde at room temperature for 15 min and formed colonies were stained with Giemsa for 30 min. Colonies containing > 20 cells were scored as positive. Significant differences between siNT (non-targeting) and specific siRNA groups or different plasmids transfection groups were analyzed via the Student’s *t*-test. The statistical difference of *p* < 0.05 was considered to indicate a statistically significant result. 

### 4.9. Mass Spectrometry

HEK293T cells were co-transfected with Flag-NSL3-3 and Myc-tagged OGT1 for 48 h, and Flag IP eluate were analyzed by mass spectrometry.

## Abbreviations

OGT	*O*-linked N-acetylglucosamine transferase
*O*-GlcNAc	*O*-linked N-acetylglucosamine
NSL	Nonspecific lethal
OGA	*O*-GlcNAcase
HAT MOF	Histone acetyltransferase Males absent on the first
